# A multi-centre, non-inferiority, randomised controlled trial to compare a cervical pessary with a cervical cerclage in the prevention of preterm delivery in women with short cervical length and a history of preterm birth – PC study

**DOI:** 10.1186/s12884-017-1393-6

**Published:** 2017-07-06

**Authors:** Bouchra Koullali, Liselotte E.M. van Kempen, Maud D. van Zijl, Christiana A. Naaktgeboren, Ewoud Schuit, Dick J. Bekedam, Maureen T.M. Franssen, Sebastiaan W.A. Nij Bijvank, Marieke Sueters, Marchien van Baal, Marjon A. de Boer, Angelo B. Hooker, Brenda B.J. Hermsen, Toon A.A.M. Toolenaar, Joost J. Zwart, David P. van der Ham, Flip W. van der Made, Federico Prefumo, Begoña Martinez de Tejada, Dimitri N.M. Papatsonis, Anjoke J.M. Huisjes, Liesbeth H.C.J. Scheepers, Marion E. van Hoorn, Tom H.M. Hasaart, Nico W.E. Schuitemaker, Karlijn C. Vollebregt, Moira A. Müller, Inge M. Evers, Marinka S. Post, Karin de Boer, Henricus Visser, Nico A. Mensing van Charante, Josje Langenveld, Nicole Y.C. Steemers, Ben W.J. Mol, Martijn A. Oudijk, Eva Pajkrt

**Affiliations:** 1 Department of Obstetrics and Gynaecology, Academic Medical Center (AMC), Amsterdam, The Netherlands; 2 Julius Center for General Practice and Health Sciences, University Medical Center Utrecht (UMCU), Utrecht, The Netherlands; 3 Department of Obstetrics and Gynaecology, Onze Lieve Vrouwe Gasthuis (OLVG) Oost, Amsterdam, The Netherlands; 4 Department of Obstetrics and Gynaecology, University Medical Center Groningen (UMCG), Groningen, The Netherlands; 5 Department of Obstetrics and Gynaecology, Isala Hospital, Zwolle, The Netherlands; 6 Department of Obstetrics and Gynaecology, Leiden University Medical Center (LUMC), Leiden, The Netherlands; 7 Department of Obstetrics and Gynaecology, Flevoziekenhuis, Almere, The Netherlands; 8 Department of Obstetrics and Gynaecology, VU Medical Center (VUmc), Amsterdam, The Netherlands; 9 Department of Obstetrics and Gynaecology, Zaans Medical Center (ZMC), Zaandam, The Netherlands; 10 Department of Obstetrics and Gynaecology, Onze Lieve Vrouwe Gasthuis (OLVG) West, Amsterdam, The Netherlands; 11 Department of Obstetrics and Gynaecology, Albert Schweitzer Hospital, Dordrecht, The Netherlands; 12 Department of Obstetrics and Gynaecology, Deventer Hospital, Deventer, The Netherlands; 13 Department of Obstetrics and Gynaecology, Martini Hospital, Groningen, The Netherlands; 14 Department of Obstetrics and Gynaecology, Sint Franciscus Gasthuis, Rotterdam, The Netherlands; 15 Department of Obstetrics and Gynaecology, Spedali Civili di Brescia and University of Brescia, Brescia, Italy; 16 Department of Obstetrics and Gynaecology, University Hospitals of Geneva and Faculty of Medicine, Geneva, Switzerland; 17 Department of Obstetrics and Gynaecology, Amphia Hospital, Breda, The Netherlands; 18 Department of Obstetrics and Gynaecology, Gelre Hospital, Apeldoorn, The Netherlands; 19 Department of Obstetrics and Gynaecology, Maastricht University Medical Centre (MUMC), Maastricht, The Netherlands; 20 Department of Obstetrics and Gynaecology, HagaZiekenhuis, Den Haag, The Netherlands; 21 Department of Obstetrics and Gynaecology, Catharina Hospital, Eindhoven, The Netherlands; 22 Department of Obstetrics and Gynaecology, Diakonessenhuis, Utrecht, The Netherlands; 23 Department of Obstetrics and Gynaecology, Spaarne Gasthuis, Haarlem, The Netherlands; 24 Department of Obstetrics and Gynaecology, Spaarne Gasthuis, Hoofddorp, The Netherlands; 25 Department of Obstetrics and Gynaecology, Meander Medical Center, Amersfoort, The Netherlands; 26 Department of Obstetrics and Gynaecology, Medical Center Leeuwarden, Leeuwarden, The Netherlands; 27 Department of Obstetrics and Gynaecology, Rijnstate Hospital, Arnhem, The Netherlands; 28 Department of Obstetrics and Gynaecology, Tergooi Hospital, Hilversum, The Netherlands; 29 Department of Obstetrics and Gynaecology, Westfries Gasthuis, Hoorn, The Netherlands; 30 Department of Obstetrics and Gynaecology, Zuyderland Hospital, Heerlen, The Netherlands; 31 Department of Obstetrics and Gynaecology, Elizabeth TweeSteden Hospital, Tilburg, The Netherlands; 32 Robinson Research Institute, School of Paediatrics and Reproductive Health, University of Adelaide, Adelaide, Australia

**Keywords:** Preterm birth, Prevention, Cerclage, Pessary, Morbidity

## Abstract

**Background:**

Preterm birth is in quantity and in severity the most important contributor of perinatal morbidity and mortality both in well- and low-resource countries. Cervical pessary and cervical cerclage are both considered as preventive treatments in women at risk for preterm birth. We aim to evaluate whether a cervical pessary can replace cervical cerclage for preventing recurrent preterm birth in women with a prior preterm birth due to cervical insufficiency or in women with a prior preterm birth and a short cervix in the current pregnancy.

**Methods/design:**

A nationwide open-label multicentre randomised clinical trial will be set up to study women with a singleton pregnancy and a prior preterm birth before 34 weeks of gestation. Women are eligible in case of previous preterm birth based on cervical insufficiency (primary intervention, <16 weeks) or in case of previous preterm birth and a short cervical length in current pregnancy ≤25 mm (secondary intervention, <24 weeks). Eligible women will be randomised to either cervical pessary or cervical cerclage. Both interventions will be removed at labour or at 36 weeks of gestational age, whatever comes first. The primary outcome will be delivery before 32 weeks. Secondary outcomes will be gestational age at birth, preterm birth rate before 24, 28, 34 and 37 weeks of gestation (overall and stratified by spontaneous or indicated delivery), premature rupture of membranes, use of tocolysis and/or corticosteroids during pregnancy, mode of delivery, maternal infections, maternal side effects, neonatal and maternal hospital admissions, and a composite of adverse perinatal outcomes including both morbidity and mortality. We assume an event rate of 20% preterm birth before 32 weeks for cerclage and use a non-inferiority margin of 10% for the cervical pessary. Using an alpha of 0.05 and power of 0.80 we need 2 groups of 200 women each.

**Discussion:**

The outcome of this study will indicate the effectiveness and the cost-effectiveness of a cervical cerclage and of a cervical pessary.

**Trial registration:**

Netherlands Trial Registry, NTR 4415. Date registered: 29th of January 2014.

**Electronic supplementary material:**

The online version of this article (doi:10.1186/s12884-017-1393-6) contains supplementary material, which is available to authorized users.

## Background

Preterm birth is defined as delivery before 37 completed weeks of gestational age (GA). The incidence of preterm birth varies between countries with a range of 5–13% and results in 15 million preterm deliveries worldwide each year. Preterm birth is a major contributor to perinatal mortality. Of all perinatal mortality, 50–70% is associated with preterm birth [1].

Approximately 75% of all preterm births occur spontaneously, starting with either contractions or preterm pre-labour rupture of membranes (PPROM). Preterm birth is the leading cause of neonatal morbidity, mostly due to respiratory immaturity, intracranial haemorrhages and infections. These conditions can result in long-term neurodevelopmental sequelae such as intellectual impairment, cerebral palsy, chronic lung disease, deafness and blindness [2]. Thus, prevention of spontaneous preterm birth remains one of the biggest challenges in obstetric care.

An important risk factor for preterm birth is a prior preterm birth. Women with a prior spontaneous preterm birth before 34 weeks have an average risk of 20% (range between 15.8% and 30.2%) of recurrence of spontaneous preterm birth before 37 weeks and 15% before 34 weeks [3, 4]. Women with a previous preterm birth before 34 weeks of gestation are usually advised to use progestagens, either 17-hydroxy progesterone caproate or vaginal progesterone, in a following pregnancy. Additionally, women with a prior preterm birth due to cervical insufficiency can be offered a primary cervical cerclage, i.e. history based cerclage. Cervical insufficiency is characterized by progressive shortening and dilatation of the cervix before 24 weeks of gestation without signs of preterm labour, and is associated with mid-trimester pregnancy loss and early preterm birth. Screening for cervical shortening by transvaginal ultrasound before 24 weeks of gestation is recommended in women with a prior preterm birth without (clear) diagnosis of cervical insufficiency in prior pregnancies. In case of a short cervix ≤25 mm before 24 weeks of gestational age, these women can be offered a secondary cervical cerclage, i.e. ultrasound indicated cerclage [5].

A cervical cerclage is a surgical procedure that involves occlusion of the cervix by means of a cervical suture or stitch which is performed under general or spinal anaesthesia proposed by Shirodkar in 1955 [6] and by McDonald in 1957 [7]. A primary cerclage is considered to be effective in the prevention of preterm birth in women with cervical insufficiency and is usually offered before 16 weeks of gestational age. The largest trial published in 1993 included 1292 women with singleton pregnancies, and showed a significant reduction in preterm birth before 33 weeks of gestation (13% versus 17%; *P* = 0.03) [8]. A meta-analysis from 2003 demonstrates that primary cervical cerclage has a significant effect in preventing spontaneous preterm birth before 34 weeks of gestation [9]. The effectiveness of a secondary cerclage has been studied in a meta-analysis from 2011. This meta-analysis found that in these women the risk of delivery before 32 weeks’ gestation was 19% in women with cerclage as compared to 30% in those without cerclage (RR 0.66 95% CI 0.48–0.91) [10].

Placement of cervical cerclages has proven to be effective in some women at risk for recurrent preterm birth. However, the disadvantage of cervical cerclage is the potential harm. Complications of cervical cerclage include PPROM, preterm labour, infection, suture displacement, and bleeding [11]. In addition, cerclage is associated with an increased risk of cervical laceration, both in nulliparous (OR 3.7, 95% CI 1.1–12.8) and multiparous women (adjusted OR 12.7, 95% CI 5.7–28.2) [12].

The cervical pessary is a soft and flexible silicone device known since 1959 when it was used in women with recurrent miscarriage [13]. Although the exact mechanism of the cervical pessary remains unknown, it has been hypothesised that the pessary relieves direct pressure on the internal cervical ostium by changing the position of the cervical canal and distributing the weight of the pregnant uterus [14]. Another possible mechanism is that the pessary might support the immunological barrier between chorioamnion-extraovular space and the vaginal microbiological flora [15]. Recently, several randomised trials showed that the cervical pessary may be potentially effective as a treatment for preterm birth prevention. The Spanish Pesario Cervical para Evitar Prematuridad (PECEP) trial from 2012 compared treatment with a pessary in women with a short cervix with expectative management and showed a significant decrease in preterm birth before 34 weeks of gestation (OR 0.19; 95% CI 0.12–0.30) and improvement of neonatal outcome (RR 0.14; 95% CI 0.04–0.39) in the intervention (pessary) group. In this study, 11% of 385 women included had at least one prior preterm birth, however, no subgroup analysis was performed for women with a previous preterm birth [15]. The same group performed a similar trial in twin pregnancies and observed a reduction in spontaneous preterm birth before 34 weeks of gestation in the pessary group (16.2% versus 25.7%; p 0.0001) [16]. Another randomised controlled trial performed by Liem et al. in 2014 comparing a pessary with no treatment in twin pregnancies showed similar results in a subgroup with short cervix [17]. Two randomised trials coordinated by the Fetal Medicine Network from the United Kingdom could not confirm these results of the pessary, both in singleton and multiple pregnancies with short cervix [18, 19]. The most frequently reported side effect of a pessary is vaginal discharge. Less common reported complications during the use of a pessary are vaginal blood loss or pelvic pain. Cervical laceration as complication is rarely seen in the use of pessary, this chance seems smaller than 0.1%.

Since a cervical pessary can be positioned in an outpatient setting, it is less expensive than placing a surgical cerclage and is therefore potentially more attractive than a cerclage. In addition, a cervical pessary is a non-invasive method contrary to a cervical cerclage which is an invasive procedure. Although both interventions have been available for over 55 years now, both interventions have been compared directly only once. A randomised study performed in 1986 in Germany in women with a prior preterm birth included 242 women and did show comparable outcomes in women using a pessary and women having cerclage (37 + 5 weeks of gestational age at delivery in the cerclage group versus 37 + 1 weeks in the pessary group, *p* value not significant) [20].

We propose to compare the cervical pessary and cervical cerclage in a head-to-head comparison and hypothesise that the use of a cervical pessary will be equally effective in preventing preterm birth as cervical cerclage. The outcome of the proposed study will indicate the relative effectiveness of cervical pessary for women with a singleton pregnancy with a prior preterm birth due to cervical insufficiency and in women with a prior preterm birth and short cervical length in current pregnancy. In addition, we will be able to compare the costs of both interventions. Since the placement of a pessary is less expensive compared to the surgical application of a cerclage, implementation of this therapy will potentially yield a major cost-reduction.

## Methods/design

### Aim, design and setting

We will perform an international randomised controlled trial under the acronym the PC Study (Pessary or Cerclage to prevent preterm delivery in women with short cervical length and a history of preterm birth; Netherlands Trial Registry NTR 4415, registered at the 29th of January 2014: website http://www.trialregister.nl/trialreg/admin/rctview.asp?TC=4415). The study will assess the effect of a cervical pessary on preterm birth rates and neonatal outcome compared to treatment with a cervical cerclage. The study is set in the Dutch Consortium for Healthcare Evaluation and Research in Obstetrics and Gynaecology - NVOG Consortium 2.0, a collaborative network of all major hospitals in The Netherlands and the Dutch Society of Obstetrics and Gynaecology (NVOG). In addition, international hospitals interested in the trial can participate in this study.

### Participants

According to local protocols, asymptomatic women with a singleton pregnancy and a prior spontaneous preterm birth before 34 weeks of gestation are offered the use of progesterone and cervical length measurements before 24 weeks of gestation. Women with a cervix ≤25 mm before 24 weeks of gestation are eligible to participate in the trial as these women would be eligible for a secondary cerclage. Additionally, women who are considered for a placement of a cerclage before 16 weeks gestation based on their obstetric history of cervical insufficiency (primary cerclage) are eligible.

### Eligibility criteria

All women with an indication for a primary or secondary cerclage, as described above, are eligible to participate in this study. Women with placenta praevia, vasa praevia, PPROM, cervical length of less than 2 mm, cervical dilation of 3 cm or more, identified major congenital or chromosomal abnormalities and women with signs of intrauterine infection will be excluded from the study. In addition, maternal age less than 18 years and inability to give informed consent are exclusion criteria.

### Procedures, recruitment, randomisation and collection of baseline data

All eligible women will be informed in brief about the clinical trial by the supervising gynaecologist or by the attending resident. Subsequently, the investigator or an authorised member of the investigational staff must explain to potential subjects the aims, methods, reasonably anticipated benefits, and potential hazards of the study. Women will be informed that their participation is voluntary and that they may withdraw consent to participate at any time. They will be informed that choosing not to participate will not affect the care they will receive.

**Fig. 1:**
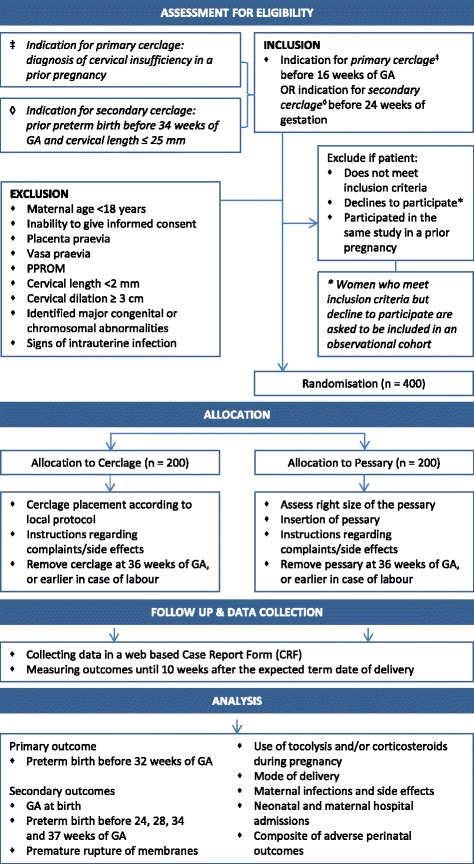
Flow diagram PC Study

Each woman must give written consent prior to randomisation. The woman will be given sufficient time to read the patient information and the informed consent form and have the opportunity to ask questions. An independent physician will be accessible for any questions the women may have. The consent form must be signed before any study-related activity can take place. A copy of the informed consent form must be given to the participating woman. Patient information is provided in Dutch and English. Women who meet all inclusion criteria but decline to participate are asked to be included in an observational cohort (see Figure 1).

Randomisation will be centrally controlled using an on-line computerised randomisation service made specifically for randomization in clinical trials, ALEA (https://nl.tenalea.net/amc/ALEA/). Centres will be able to access the randomisation service 24 h/day. Eligible women will be randomized in a 1:1 ratio to cervical cerclage and pessary (see Figure 1). Randomisation will be stratified by indication for type of cerclage (primary or secondary) and centre (to prevent any imbalance between groups in aspects of maternal or neonatal care that may differ between centres). We will apply block randomisation with a variable block size of 4 and 6. Due to the type of interventions this study will not be blinded.

Baseline characteristics (e.g. patient demographics, obstetric and medical history), details of delivery, maternal and neonatal assessments during pregnancy or post-partum will be recorded into a web-based Case Report Form (CRF) that is accessible through a closed part of a web-secured database (see Figure 1). We included core outcomes for preterm birth in the CRF [21]. The CRF can be found in Additional file 1.

### Confidentiality and data security

Initials of participants as well as year of birth are recorded in the electronic database. Linking personal data with randomisation number can only be done in the local clinics. Each participating clinic receives a login name and password to gain access to the web-secured database. The access is restricted to the database of the clinic to which the password and login name belongs. Full access to the entire database is possible to some members of the research staff, but has to be requested via the trial bureau and data manager of the NVOG Consortium 2.0.

### Intervention

Eligible women will be randomly allocated to receive either a cervical cerclage or a cervical pessary (Arabin® pessary). Both will be placed before 24 weeks, or before 16 weeks in case of a primary intervention. Women allocated to a cervical cerclage will be receiving the intervention according to local protocol. Women allocated to a cervical pessary will receive a simple vaginal examination to assess which size pessary fits best. It is important that the pessary is placed by a care giver with expertise to ensure careful placement of the pessary. In case of complaints, (vaginal) examination of the patient is advised to reposition the pessary or to replace the pessary with another size if necessary. Both interventions will stay in place until 36 weeks of gestation or until delivery, whatever comes first. If recurrent or persistent blood loss, premature rupture of the membranes or contractions occur during the use of a pessary, the pessary should be removed. Further management will be according to the national guideline on prevention of preterm birth and local protocols (see Figure 1).

### Outcome measures

#### Primary outcome measure

The primary outcome will be delivery before 32 weeks of gestation.

#### Secondary outcome measures

Secondary outcomes will be time from intervention to delivery, gestational age at birth, preterm birth rate before 24, 28, 34 and 37 weeks of gestation (overall and stratified by spontaneous or indicated delivery), premature rupture of membranes, use of tocolysis and/or corticosteroids during pregnancy, mode of delivery, maternal infections, maternal side effects and both neonatal and maternal hospital admissions. Perinatal outcome will be assessed through a composite of adverse perinatal outcome. This composite outcome contains chronic lung disease, intraventricular haemorrhage (IVH) > grade II, periventricular leucomalacia (PVL) > grade I, necrotising enterocolitis (NEC) > stage I, retinopathy of prematurity (ROP), patent ductus arteriosus (PDA), treated seizures, early and late onset sepsis, neonatal meningitis, (intra-partum) stillbirth, death before discharge from the nursery. The definitions of these outcomes can be found in Table 1. All components of the composite outcome will also be assessed individually.

**Table 1:** Definitions of secondary outcome measures

Outcome	Defined as:
Maternal infections	Two measurements of maternal temperature above 37,8 degrees Celsius at a one hour interval and a maternal pulse >100 beats per minute requiring treatment with antibiotics
Maternal side effects	Vaginal discharge, bleeding, discomfort, dyspareunia, cervical laceration
Chronic lung disease	Babies born before 32 weeks: need for >30% oxygen, with or without positive pressure ventilation or continuous positive pressure at 36 weeks postmenstrual age, or discharge (whichever comes first). Babies born after 32 weeks: need for >30% oxygen with or without positive pressure ventilation or continuous positive pressure at 56 days postnatal age, or discharge (whichever comes first).
IVH > grade II	Haemorrhage in the germinal matrix, ventricles, or cerebral parenchyma; observed on ultrasound examination or MRI
PVL > grade I	Periventricular lucency in the white matter
NEC > stage I	Defined as the presence of the characteristic clinical features of abdominal distension, with or without rectal bleeding, and abdominal radiographic finding associated with pneumatosis intestinalis
Early sepsis	If prior to or at 72 h of life the infant had an infection marked by positive blood, CSF, or urine (catheterised or suprapubic) cultures with or without suspicious clinical findings of infection on physical examination.
Late sepsis	If after 72 h of life the infant had an infection marked by positive blood, CSF, or urine (catheterised or suprapubic) cultures with or without suspicious clinical findings of infection on physical examination OR if there is clinical evidence of cardiovascular collapse or an unequivocal X-ray confirming infection and often cardiovascular decomposition
Neonatal meningitis	Suspected or proven (caused by any pathogen)

In addition, a cost-effectiveness analysis will be performed that will be reported separately from the primary report on the randomised trial.

### Follow up of women and infants

All details of delivery, maternal assessments and admittance during pregnancy will be recorded in an electronic case record form that will be accessible through the web-secured database. In case of admittance of a newborn, details of admittance will also be recorded. The outcome measures, when applicable, will be measured until 10 weeks after the expected term date of delivery.

The possibilities to perform long term follow-up will be assessed and planned, depending on the outcomes of the primary study and granted funding. Permission to approach women for follow-up research will be asked by the initial informed consent.

### Statistical issues

#### Sample size

We plan to evaluate the non-inferiority of a cervical pessary as compared to cervical cerclage. We assume an event rate of 20% for the primary outcome, i.e. delivery before 32 weeks, for cerclage based on current literature. We will use a non-inferiority margin of 10%. If the event rate is 20% for the primary outcome in the cerclage arm, this is equivalent to saying that a pessary is non-inferior to cerclage when the upper limit of the 95% confidence interval of the event rate of the primary outcome in the pessary group is less than 30%. Using a one-sided alpha of 0.05 and power of 0.80 we need 2 groups of 200 women each.

#### Data analysis

Data will initially be analysed according to the intention to treat method. The primary outcome will be assessed investigating whether the prevalence of the primary outcome, birth before 32 weeks, is not more than 10% higher in the pessary group compared to the cerclage group. Non-inferiority will be concluded when the upper end of a one-sided 95% confidence interval for the risk difference between the prevalence of the primary outcome in the pessary group and the cerclage group is less than 10%.

The secondary outcome time from intervention to delivery will be evaluated by Cox proportional hazard analysis and Kaplan-Meier estimates and plots, with account for different durations of gestation at entry and stratification by indication for intervention (and centre when data allows), and will be tested with the log rank test. Secondary dichotomous outcome measures will be assessed by calculating absolute and relative risks, along with 95% confidence intervals. Differences in continuous outcomes between both strategies will be assessed using a linear mixed model.

In all analyses, stratification by centre will be accounted for with a random intercept for each centre, and by adding the cerclage indication as a covariate to the log-binomial or linear mixed models. If these models fail, A Cochran–Mantel–Haenszel (CMH) approach will be used to take into account that randomisation was stratified on indication for cerclage (and centre, if the data allows). Such a stratified analysis of the estimates the risk differences from each covariate subgroup and uses CMH weights to estimate treatment difference and its standard error. When appropriate, numbers needed to treat will be calculated.

#### Subgroup analysis

We pre-specify three subgroup analyses based on: (1) the indication of the type of cerclage to investigate the effectiveness of the pessary compared to a primary and secondary cerclage separately, (2) the number of previous preterm births (overall and separately for indication for primary or secondary intervention) in which we distinguish those with one previous preterm birth from those with two or more previous preterm births and (3) cervical length ≤15 mm and >15 mm in women with an indication for secondary intervention. Subgroup effects will be investigated for the primary outcome, preterm delivery before 32 weeks of gestation, and for the composite of perinatal outcome. Subgroup effects will be assessed by including an interaction term between the subgrouping variable and treatment allocation as covariate to the regression model. Afterwards, a stratified subgroup analysis will be performed to study the effect of treatment in different strata of the subgroups.

To evaluate the potential of each of the strategies, we will also perform a per protocol analysis, taking into account only those cases that were treated according to protocol.

### Safety

#### (Serious) Adverse Event ([S]AE)

All AEs reported spontaneously by the subject or observed by the investigator or his staff will be recorded. All SAEs will be reported through the web portal *ToetsingOnline* to the accredited medical ethics committee (MEC) that approved the protocol.

#### Interim safety review

Safety reviews will be performed after all outcomes of 110 inclusions are available for analysis, and thereafter as determined necessary by the independent data and safety monitoring board (DSMB). The DSMB will be unblinded before making recommendations, but the researchers are to remain blinded. An extra meeting will be planned if indicated by the safety review. The data and safety monitoring committee can advise to stop the study for safety reasons.

## Discussion

To our knowledge there are no other registered on-going trials comparing the effect of a cervical pessary and a cervical cerclage in women at high risk for preterm birth.

When the pessary was first described in 1959, it was used in women with recurrent late miscarriages and possible cervical insufficiency [13]. The largest randomised controlled trial so far shows no difference between cervical cerclage and pessary in women with previous spontaneous preterm birth and an indication for a cerclage [20]. In addition, the recent PECEP study, a study which compared treatment with a pessary in women with a short cervix with expectative management, included 11% women with at least one prior preterm birth. This study showed an overall significant decrease in preterm birth in the intervention (pessary) group (spontaneous delivery before 34 weeks 12 (6%) in the pessary group vs. 51 (27%) in de control group; OR 0.18; 95% CI 0.08–0.37; *p* < 0·0001), however, no subgroup analysis was performed for women with a previous preterm birth [15]. There are clues that a cervical pessary might be as effective as a cerclage in the prevention of preterm birth, however, large recent randomized controlled trials with information on the effectiveness of a pessary in women with a previous preterm birth are lacking.

A cervical cerclage is considered to be effective in the prevention of preterm birth in women with cervical insufficiency and/or short cervix during pregnancy with a previous preterm birth. However, it is associated with serious risks such as premature rupture of membranes, premature contractions, cervical and/or uterine infections, vaginal bleeding and cervical laceration [11, 12]. Although the chance of these complications occurring is indeed low, the impact on the course of the pregnancy is major. Additionally, cervical cerclage is a surgical intervention which is usually performed under general anaesthesia, and as such is at risk of surgical complications. A cervical pessary is a non-invasive intervention and can be placed in an outpatient setting. In addition, severe complications related to cerclage are considered to occur more often compared to complications related to the use of pessary. This makes a pessary more attractive as intervention, however, the effect on preterm birth and neonatal outcome should be addressed first.

The outcome of this study will determine whether treatment with a cervical pessary can replace a cerclage to prevent preterm birth in women at high-risk for preterm delivery.

## Additional files

Additional file 1: Case Report Form (CRF) PC Study. (DOCX 155 kb)

Additional file 2: SPIRIT checklist for reporting randomised trials for the PC Study. (DOC 120 kb)

## Abbreviations

AE: Adverse event
CMH: Cochran–Mantel–Haenszel
CRF: Case report form
DSMB: Data and safety monitoring board
GA: Gestational age
IVH: Intraventricular haemorrhage
MEC: Medical ethics committee
NEC: Necrotising enterocolitis
NVOG: Dutch society of obstetrics and gynaecology
PDA: Patent ductus arteriosus
PECEP: Pesario cervical para evitar prematuridad
PPROM: Preterm pre-labour rupture of membranes
PVL: Periventricular leukomalacia
ROP: Retinopathy of prematurity
SAE: Serious adverse event
