# Supplementary material for: A multi-centre, non-inferiority, randomised controlled trial to compare a cervical pessary with a cervical cerclage in the prevention of preterm delivery in women with short cervical length and a history of preterm birth – PC study
Source: BMC Pregnancy Childbirth. 2017 Jul 6;17:215. doi: 10.1186/s12884-017-1393-6 (PMC5501372; doi:10.1186/s12884-017-1393-6)
Supplement: Supplementary file 1 — Case Report Form (CRF) PC Study. (DOCX 155 kb) [file 12884_2017_1393_MOESM1_ESM.docx]

PC study

Confidential

Case report form

Compare a cervical pessary with cervical cerclage in the prevention of preterm birth in asymptomatic women with a singleton pregnancy and with previous preterm birth (<34 weeks) either with a short cervical length (≤25 mm) or with a history of multiple preterm births

Case number: ⬜⬜⬜-⬜⬜⬜

*This is a CRF hand-out for the purpose of support for the PC Study website.*

CRF data entry, information and randomisation:

http://www.studies-obsgyn.nl/pc

Coordinating researcher: B. Koullali

Principal investigator: E. Pajkrt, gynecologist

**Table of contents**

1. **General……………………………………………………………………………………… 3**
   1. General information……………………………………………………………........... 3
   2. Inclusion criteria………………………………………………………………….......... 4
   3. Exclusion criteria………………………………………………………………………. 4
2. **Randomization.……………………………………………………………………………. 5**
3. **Demographics……………………………………………………………………………... 6**
4. **Medical history…………………………………………………………………………….. 7**
5. **Obstetric history…………………………………………………………………………... 8**
6. **Current pregnancy………………………………………………………………………... 9**
   1. Start of pregnancy……………………………………………………………………... 9
   2. Laboratory results at study entry…………………………………………………….. 9
   3. Transvaginal ultrasound at study entry.…………………………………………….10
   4. Medical interventions during pregnancy……………………………………………10
7. **Intervention……………………………………………………………………………….. 11**
8. **Complications and admissions after randomization……………………………… 14**8.1. Complications after randomization………………………………………………… 14
   8.2. Admissions after randomization……………………………………………………. 15
9. **Delivery……………………………………………………………………………………. 16**
   1. Onset of labor/induction……………………………………………………………. 16
   2. Delivery………………………………………………………………………………... 16
10. **Neonatal data post-partum…………………………………………………………….. 17**
11. **Post-partum admission………………………………………………………………… 18**
    1. Maternal admission…………………………………………………….................... 18
    2. Neonatal admission…………………………………………………………………. 19
12. **Neonatal diagnosis……………………...………………………………………………. 21**
13. **Serious Adverse Event (SAE)………………………………………………………….22**
14. **End of Study……………………………………………………………………………… 23**

**TO BE FILLED OUT AT STUDY ENTRY:**

1. **General**
   1. **General information**

Clinic: ⬜⬜⬜

Case number: ⬜⬜⬜ - ⬜⬜⬜ (clinic - case)

Date of birth: ⬜⬜-⬜⬜-⬜⬜ (dd-mm-yy)

Estimated due date ⬜⬜-⬜⬜-⬜⬜ (dd-mm-yy)

**Intervention**

O Primary intervention (<16 weeks)

O Secondary intervention (16-24 weeks)

- 1. **Inclusion criteria**

| Inclusion criteria | |  | No | Yes |
| --- | --- | --- | --- | --- |
| 1. | Singleton pregnancy | | O | O |
| 2. | History of preterm birth before 34 weeks of gestation | | O | O |
| 3. | Cervical length ≤ 25mm < 24 weeks of GA  OR  Indication for primary cerclage < 16 weeks of GA* | | O | O |

* The indication for primary cerclage can be determined by local protocol

- 1. **Exclusion criteria**

| Maternal/Fetal Exclusion criteria | |  | No | Yes |
| --- | --- | --- | --- | --- |
|  | Maternal age less than 18 years | | O | O |
|  | Inability to give informed consent | | O | O |
|  | Placenta praevia | | O | O |
|  | Vasa praevia | | O | O |
|  | Ruptured membranes | | O | O |
|  | Cervical length < 2 mm | | O | O |
|  | Cervical dilatation ≥3cm | | O | O |
|  | Identified major congenital abnormalities# | | O | O |
|  | Women with clinical signs of chorioamnionitis or signs of intra uterine infection# | | O | O |

**^#^** **^Major fetal abnormalities are defined as those that are lethal or require intensive prenatal care or postnatal surgery. Women having signs of chorioamnionitis or signs of intra uterine infection, defined as a temperature > 37,8 degrees Celsius, maternal tachycardia > 100 beats per minute and fetal tachycardia > 160 beats per minute, without any other focus of infection.^**

1. **Randomization**

**Date of randomization:** ⬜⬜-⬜⬜-⬜⬜ (dd-mm-yy)

**Randomization result:**

O Pessary

O Cerclage

O No randomization (observational cohort)

|

In case of no consent for randomization but consent for observational cohort, please fill in the following five questions:

Estimated due date ⬜⬜-⬜⬜-⬜⬜ (dd-mm-yy)

Intervention O Pessary O Cerclage O None

*Child*

Live birth O Yes

O Termination of pregnancy

O No, deceased before labor

O No, deceased during labor

Date of birth or date of diagnosis of death ⬜⬜-⬜⬜-⬜⬜ (dd-mm-yy)

Gender: O Boy O Girl

**TO BE FILLED OUT AFTER RANDOMISATION:**

1. **Demographics**

**Ethnicity:**

O Caucasian

O Indian/ Pakistani/ Bangladesi

O Afro-Caribbean (Antillen, Suriname –creool)

O Hindu, Caribbean (Suriname – Hindu)

O African (sub-Sahara)

O Middle-East + North Africa (Turkish and Moroccan)

O Asian

O Other

O Unknown

**Highest finished education mother:**

O Primary school (basisschool)

O Secondary school (havo, VWO, gymnasium)

O Lower professional school (VMBO)

O Medium professional school (MBO)

O Higher professional school (HBO – post HBO)

O University (WO)

O Unknown

1. **Medical history**

**Diseases:**

Diabetes Mellitus O no O yes

Hypertension O no O yes

Renal insufficiency O no O yes

Inflammatory bowel disease O no O yes

Thrombophilia O no O yes

Hyperthyroid disease O no O yes

Depression O no O yes

**Risk factors for preterm delivery:**

Conisation O no O yes O unknown

LLETZ O no O yes O unknown

Uterus anomaly O no O yes O unknown

Known uterine surgery O no O yes O unknown

Smoking O no O yes O quitted in first trimester O unknown | |

If yes or quitted,

Amount: ⬜⬜ sig/day

Urinary tract infections last year

O no O yes O unknown

|

If yes,

Amount: ⬜⬜ last year (total)

1. **Obstetric history**

Gravidity ⬜⬜ ; Parity ⬜⬜; Miscarriage/abortion/ectopic pregnancy* ⬜⬜

* < 16 weeks |

Was there any curettage?

O no O yes O unknown

1 = spontaneously

2 = vacuum

3 = forcipal extraction

4 = caesarian

-1 = unknown

3

1 = spontaneously

2 = induction

3 = caesarian (primary)

-1 = unknown

0 = not applicable

1 = primary cerclage

2 = secondary cerclage

3 = tertiary cerclage

4 = pessary

-1 = unknown

GA: weeks + days

XX/’XX

Date of birth

Month/Year GA Start Route Intervention in pregnancy

1. ⬜⬜/⬜⬜ ⬜⬜+⬜ ⬜ ⬜ ⬜

2. ⬜⬜/⬜⬜ ⬜⬜+⬜ ⬜ ⬜ ⬜

3. ⬜⬜/⬜⬜ ⬜⬜+⬜ ⬜ ⬜ ⬜

4. ⬜⬜/⬜⬜ ⬜⬜+⬜ ⬜ ⬜ ⬜

5. ⬜⬜/⬜⬜ ⬜⬜+⬜ ⬜ ⬜ ⬜

6. ⬜⬜/⬜⬜ ⬜⬜+⬜ ⬜ ⬜ ⬜

7. ⬜⬜/⬜⬜ ⬜⬜+⬜ ⬜ ⬜ ⬜

8. ⬜⬜/⬜⬜ ⬜⬜+⬜ ⬜ ⬜ ⬜

9. ⬜⬜/⬜⬜ ⬜⬜+⬜ ⬜ ⬜ ⬜

10. ⬜⬜/⬜⬜ ⬜⬜+⬜ ⬜ ⬜ ⬜

11. ⬜⬜/⬜⬜ ⬜⬜+⬜ ⬜ ⬜ ⬜

12. ⬜⬜/⬜⬜ ⬜⬜+⬜ ⬜ ⬜ ⬜

13. ⬜⬜/⬜⬜ ⬜⬜+⬜ ⬜ ⬜ ⬜

14. ⬜⬜/⬜⬜ ⬜⬜+⬜ ⬜ ⬜ ⬜

1. **Current pregnancy**
   1. ***Start of pregnancy***

Mode of conception:

Ο Spontaneous

Ο IUI

Ο Ovulation induction

Ο IVF

Ο ICSI

Height: ⬜⬜⬜ cm (140-210)

Weight before pregnancy ⬜⬜⬜ kg (35-200)

Estimated due date ⬜⬜-⬜⬜-⬜⬜ (dd-mm-yy)

- 1. ***Laboratory results at study entry***

Nugent score (first trimester) O no O yes O unknown

|

If yes, score:

O < 4 O 4-7 O ≥ 7

If Nugent score ≥ 4, treatment:

Ο yes

Ο no

Ο unknown

Vaginal swab (most recent) O no O yes O unknown

|

If yes, result:

⬜ negative

⬜ positive for GBS

⬜ positive for E.coli

⬜ trichomonas

⬜ candida

⬜ other

Fibronectin test O negative O positive O not performed

|

If applicable, quantitative result: ⬜⬜⬜ ng/mL (999 if result is >500 ng/mL)

- 1. ***Transvaginal Ultrasound at study entry***

Was a transvaginal ultrasound performed at randomization?

O no O yes

|
If yes;

Date of cervical length measurement ⬜⬜-⬜⬜-⬜⬜ (dd-mm-yy)

Cervical length at randomization ⬜⬜ mm (0 to 80)

Funneling O no O yes O unknown

Sludge O no O yes O unknown

- 1. ***Medical interventions during pregnancy***

Did the patient receive progesterone O no O yes O unknown

|

If yes:

Ο Uterogestan

Ο Proluton

Ο Unknown

Did the patient receive tocolytics at any time during pregnancy?

O no O yes O unknown

Did the patient receive corticosteroids at any time during pregnancy?

O no O yes O unknown

Did the patient receive magnesium sulfate for neuroprotection?

O no O yes O unknown

1. **Intervention**

Randomization result*:

O Pessary O Cerclage

** When randomized to pessary, please fill in the questions ‘if randomized to pessary’ only. When randomized to cerclage, please fill in the questions ‘if randomized to cerclage’ only.*

**If randomized to pessary**

Did patient receive a pessary: O no O yes

|

Ο placement failed, cervical length too small

Ο pessary size not in stock

Ο woman refused pessary placement

Ο delivered before placement

Ο patient preferred a cerclage

Ο doctor preferred a cerclage

Ο unknown

Date of placing pessary ⬜⬜-⬜⬜-⬜⬜ (dd-mm-yy)

Size pessary

O Small 65x25x32

O Medium (standard) 70x25x32

O Large 70x25x35

When was the pessary removed?

Date: ⬜⬜ - ⬜⬜ - ⬜⬜ (dd-mm-yy)

Why was the pessary removed?

# ⬜ GA > 36 weeks ⬜ discomfort

⬜ excessive discharge

⬜ patient preferred a cerclage

⬜ pessary fell out

⬜ (P)PROM

⬜ vaginal blood loss

⬜ contractions/labor

⬜ require delivery for other reasons

# If the pessary was removed, was it replaced?

O no O yes

|

If yes,

Date: ⬜⬜ - ⬜⬜ - ⬜⬜ (dd-mm-yy)

|

If no, was it replaced by a cerclage?

O no O yes O unknown

|

If yes, Date: ⬜⬜ - ⬜⬜ - ⬜⬜ (dd-mm-yy)

Material used for cerclage

Ο braided polyfilament (tape)

Ο monofilament

Technique used for cerclage

Ο MacDonald

Ο Shirodkar

Why was the second pessary (or cerclage) removed?

⬜ GA > 36 weeks
⬜ discomfort

⬜ excessive discharge

⬜ patient preferred a cerclage

⬜ pessary fell out

⬜ (P)PROM

⬜ vaginal blood loss

⬜ contractions/labor

⬜ require delivery for other reasons

When was the second pessary (or cerclage) removed?

Date: ⬜⬜ - ⬜⬜ - ⬜⬜ (dd-mm-yy)

**If randomization to cerclage:**

Did patient receive a cerclage: O no O yes

|

Ο placement failed cervical length too small.

Ο woman refused cerclage placement

Ο delivered before placement

Ο patient preferred a pessary

Ο doctor preferred a pessary

Ο unknown

Date of placing cerclage ⬜⬜-⬜⬜-⬜⬜ (dd-mm-yy)

Material used for cerclage Ο braided polyfilament (tape)

Ο monofilament

Technique used for cerclage Ο MacDonald

Ο Shirodkar

When was the cerclage removed?

Date: ⬜⬜ - ⬜⬜ - ⬜⬜ (dd-mm-yy)

# Why was the cerclage removed?

# ⬜ GA > 36 weeks ⬜ discomfort

⬜ excessive discharge

⬜ patient preferred a pessary

⬜ (P)PROM

⬜ vaginal blood loss

⬜ contractions/labor

⬜ require delivery for other reasons

If the cerclage was removed, was it replaced?

O no O yes O unknown

|

If yes, Date: ⬜⬜ - ⬜⬜ - ⬜⬜ (dd-mm-yy)

Material used for cerclage

Ο Braided polyfilament

Ο Monofilament

Technique used for cerclage

Ο MacDonald

Ο Shirodkar

|

If no, was it replaced by a pessary?

O no O yes O unknown

|

If yes, Date: ⬜⬜ - ⬜⬜ - ⬜⬜ (dd-mm-yy)

Why was the second cerclage (or pessary) removed?

# ⬜ GA > 36 weeks ⬜ discomfort

⬜ excessive discharge

⬜ patient preferred a cerclage

⬜ patient preferred a pessary

⬜ (P)PROM

⬜ vaginal blood loss

⬜ contractions/labor

⬜ require delivery for other reasons

When was the second cerclage (or pessary) removed?

Date: ⬜⬜ - ⬜⬜ - ⬜⬜ (dd-mm-yy)

1. **Complications and admissions after randomization**
   1. ***Complications after randomization***

Treated genital tract infection O no O yes O unknown

Treated urinary tract infection O no O yes O unknown

Ruptured membranes <36 weeks O no O yes O unknown

|

If yes, Date:
⬜⬜ - ⬜⬜ - ⬜⬜ (dd-mm-yy)

Excessive vaginal discharge O no O yes O unknown

Vaginal blood loss O no O yes O unknown
*not related to placing pessary/cerclage*

Pain O no O yes O unknown

*not related to placing pessary/cerclage*

Chorioamnionitis

*Requires 2 of the following: fever, fundal tenderness, maternal tachycardia, fetal tachycardia, foul-smelling amniotic fluid*

O no O yes O unknown

- 1. ***Admissions after randomization***

1= Threatened

preterm birth

2= PE

3= IUGR

4= Complication of

placing cerclage

5= Decreased fetal

movement

6= Labor/induction

7= PPROM

8= Vaginal bleeding

9= Other

10=unknown

***Fill in the details for number of admissions:***

1= Ward

2= Medium care

3= Intensive care

| **Admission**  **number** | **Date of admission to hospital** (dd-mm-yy) | **Date of discharge from hospital** (dd-mm-yy) | **Type of admission** | **Primary indication for admission** |
| --- | --- | --- | --- | --- |
| 1 | ⬜⬜ - ⬜⬜ - ⬜⬜ | ⬜⬜ - ⬜⬜ - ⬜⬜ |  |  |
| 2 | ⬜⬜ - ⬜⬜ - ⬜⬜ | ⬜⬜ - ⬜⬜ - ⬜⬜ |  |  |
| 3 | ⬜⬜ - ⬜⬜ - ⬜⬜ | ⬜⬜ - ⬜⬜ - ⬜⬜ |  |  |
| 4 | ⬜⬜ - ⬜⬜ - ⬜⬜ | ⬜⬜ - ⬜⬜ - ⬜⬜ |  |  |
| 5 | ⬜⬜ - ⬜⬜ - ⬜⬜ | ⬜⬜ - ⬜⬜ - ⬜⬜ |  |  |
| 6 | ⬜⬜ - ⬜⬜ - ⬜⬜ | ⬜⬜ - ⬜⬜ - ⬜⬜ |  |  |
| 7 | ⬜⬜ - ⬜⬜ - ⬜⬜ | ⬜⬜ - ⬜⬜ - ⬜⬜ |  |  |
| 8 | ⬜⬜ - ⬜⬜ - ⬜⬜ | ⬜⬜ - ⬜⬜ - ⬜⬜ |  |  |

1. **Delivery**
   1. ***Onset of labor/induction***

Start date labor onset: ⬜⬜-⬜⬜-⬜⬜ (dd-mm-yy)

Onset of labor:

O Spontaneous contractions

O Spontaneous rupture of membranes

If induction, reason: ⬜ maternal indication

⬜ fetal indication

⬜ ruptured membranes

⬜ elective

O Induction 🡪

O Primary C-Section

- 1. ***Delivery***

##### Birth child: Date ⬜⬜-⬜⬜-⬜⬜ (dd-mm-yy) Time ⬜⬜:⬜⬜ (hh-mm)

Route of delivery* O Spontaneously

O Vacuum extraction

O Forcipal extraction

O Caesarean section

** If route of delivery is spontaneous, please skip questions ‘indication vacuum/forceps’ and ‘indication Ceasarean section’. If route of delivery is vacuum/forcipal extraction, skip only ‘indication Ceasarean section’. If route of delivery is Ceasarean section, please skip ‘indication vacuum/foceps’.*

Indication vacuum/ forceps? ⬜ Fetal distress

⬜ Maternal indication

⬜ Failure to progress in second stage

Indication Caesarean section? ⬜ Failure to progress, first stage

⬜ Failure to progress, second stage

⬜ Failed instrumental delivery

⬜ Fetal distress

⬜ Elective reason

⬜ Maternal indication

Presentation at birth O Cephalic

O Breech

O Transverse

Did mother require antibiotics in labor for treatment of suspected infection (not prophylaxis)? O no O yes O unknown

Treated postpartum endometritis or pelvic infection? O no O yes

1. **Neonatal data post-partum**

*Child*

Live birth O Yes

O Termination of pregnancy

O No, deceased before labor

O No, deceased during labor

Date of diagnosis of death ⬜⬜-⬜⬜-⬜⬜ (dd-mm-yy)

Gender: O Boy O Girl

Apgar-scores:
1 min ⬜⬜ (0–10)

5 min ⬜⬜ (0–10)

Weight at birth ⬜⬜⬜⬜ gr. (0 – 6000)

Umbilical cord measurements: (pH: 6.00-7.70; BE: -30.0-+10.0; unknown: -1)

Art. pH ⬜ ⬜.⬜⬜ Art. BE ⬜ ⬜⬜.⬜

Ven. pH ⬜ ⬜.⬜⬜ Ven. BE ⬜ ⬜⬜.⬜

Fetal anomaly O no O yes

|

⬜ Ambiguous genitalia female genotype

⬜ Ambiguous genitalia male genotype

⬜ Anencephaly

⬜ Ascites

⬜ Bowel atresia / volvulus / obstruction

⬜ Cleft lip

⬜ Cleft palate

⬜ Congenital diaphragmatic hernia

⬜ Cyanotic congenital heart disease

⬜ Down syndrome / trisomy 21

⬜ Gastroschisis

⬜ Hydrocephalus

⬜ Hypospadias

⬜ Limb reduction defect

⬜ Meningomyelocele / spina bifida

⬜ Myocarditis / dilated cardiomyopathy

⬜ Omphalocele

⬜ Pericardial effusion

⬜ Postaxial polydactyly

⬜ Renal anomaly

⬜ Suspected chromosomal disorder

⬜ VSD

⬜ Other

1. **Postpartum Admission**

Were mother or child admitted directly postpartum?*

O No, there was no admission – END OF CRF

O Yes, maternal admission only ** Please fill in ‘Maternal admission’ only.*

O Yes, neonatal admission only ** Please fill in ‘Neonatal admission’ only.*

O Yes, maternal and neonatal admission

If yes: O maternal indication O neonatal indication O mother and child

** Please fill in both ‘Maternal and Neonatal admission’*

- 1. **Maternal Admission**

**Admission #1**

Type of admission O Ward

O Medium care

O Intensive care

Discharge to O Home Complete “Date of final discharge to home”

O Other ward ------- Transfer date ⬜⬜-⬜⬜-⬜⬜ (dd-mm-yy)

O Other hospital

**Admission #2**

Type of admission O Ward

O Medium care

O Intensive care

Discharge to O Home Complete “Date of final discharge to home”

O Other ward ------- Transfer date ⬜⬜-⬜⬜-⬜⬜ (dd-mm-yy)

O Other hospital

**Admission #3**

Type of admission O Ward

O Medium care

O Intensive care

Discharge to O Home Complete “Date of final discharge to home”

O Other ward ------- Transfer date ⬜⬜-⬜⬜-⬜⬜ (dd-mm-yy)

O Other hospital

Maternal death O No O Yes

|

date ⬜⬜-⬜⬜-⬜⬜ (dd-mm-yy)

time ⬜⬜:⬜⬜ (hh:mm)

*Date of final discharge to home* ⬜⬜-⬜⬜-⬜⬜ (dd-mm-yy)

- 1. **Neonatal Admission**

**Admission #1**

Type of admission O Ward

O Medium care

O Neonatal intensive care unit

Discharge to O Home Complete “Date of final discharge to home”

O Other ward------ Transfer date ⬜⬜-⬜⬜-⬜⬜ (dd-mm-yy)

O Other hospital

**Admission #2**

Type of admission O Ward

O Medium care

O Neonatal intensive care unit

Discharge to O Home Complete “Date of final discharge to home”

O Other ward------ Transfer date ⬜⬜-⬜⬜-⬜⬜ (dd-mm-yy)

O Other hospital

**Admission #3**

Type of admission O Ward

O Medium care

O Neonatal intensive care unit

Discharge to O Home Complete “Date of final discharge to home”

O Other ward------ Transfer date ⬜⬜-⬜⬜-⬜⬜ (dd-mm-yy)

O Other hospital

Neonatal death before 28 days? O No O Yes

|

date ⬜⬜-⬜⬜-⬜⬜ (dd-mm-yy)

time ⬜⬜:⬜⬜ (hh:mm)

Neonatal death after 28 days? O No O Yes

|

date ⬜⬜-⬜⬜-⬜⬜ (dd-mm-yy)

time ⬜⬜:⬜⬜ (hh:mm)

*Date of final discharge to home* ⬜⬜-⬜⬜-⬜⬜ (dd-mm-yy)

1. **Neonatal diagnosis**

*Diagnosis as reported in the neonatal discharge letter of the pediatrician*

| Chronic lung disease | O No | O Yes, no therapy  O Yes, only intubation/CPAP  O Yes, intubation/CPAP and surfactant |
| --- | --- | --- |
| Necrotizing Enterocolitis (NEC)*^2^ | O No | O Stage 1 O Stage 2 O Stage 3 |
| Intracerebral haemorrhage (IVH)*^3^ | O No | O Grade 1 O Grade 2 O Grade 3  O Grade 4 O Grade unknown |
| Periventricular leukomalacia (PVL)*^4^ | O No | O Grade 1 O Grade 2 O Grade 3  O Grade 4 O Grade unknown |
| Retinopathy of Prematurity (ROP) | O No | O Yes, no therapy  O Yes, conservative treatment (e.g. oxygen or medical therapy)  O Yes, requiring surgery |
| Patent ductus arteriosus (PDA) | O No | O Yes, no therapy  O Yes, conservative treatment (e.g. medical therapy)  O Yes, requiring surgery |
| Treated seizures | O No | O Yes |
| Early neonatal sepsis (<72 hours)*^5^ | O No | O Suspected  O Proven with positive culture |
| Late neonatal sepsis (>72 hours)*^6^ | O No | O Suspected  O Proven with positive culture |
| Neonatal meningitis | O No | O Suspected  O Proven with positive culture |

* Defined as:

1. infants with oxygen dependency at either 28 days of life or 36 weeks’ gestation
2. Necrotizing Enterocolitis (NEC) > stage 1: the presence of the characteristic clinical features of abdominal distention, with or without rectal bleeding, and abdominal radiographic finding associated with pneumatosis intestinalis (this last finding is an abnormal gas pattern with dilated loops consistent with ileus)
3. intracerebral haemorrhage grade III or IV: IVH with ventricular dilation or parenchymal extension, confirmed by MRI, sonogram or CT scan
4. Periventricular lucency in the white matter.
5. If prior to or at 72 hours of life the infant had an infection marked by positive blood, CSF, or urine (catheterized or suprapubic) cultures with or without suspicious clinical findings of infection on physical examination.
6. If after 72 hours of life the infant had an infection marked by positive blood, CSF, or urine (catheterized or suprapubic) cultures with or without suspicious clinical findings of infection on physical examination.
   OR
   If there is clinical evidence of cardiovascular collapse or an unequivocal X-ray confirming infection and often cardiovascular decomposition
7. **Serious Adverse Event**

# In this pregnancy has there been a Serious Adverse Event (SAE)?

O no O yes

|
 ⬜ Perinatal death

⬜ Maternal death

⬜ Maternal hospitalization or prolongation of existing inpatients’ hospitalization (not related to delivery or threatened preterm birth)

# In this pregnancy has there been an Adverse Event (AE)?

O no O yes

|
 ⬜ Preterm labor (before AD 34^0/7^)

⬜ Severe neonatal morbidity (NICU admission)

⬜ PPROM due to placing cerclage (within 72 hours)

⬜ Necrotic cervix due to pessary

| **14. End of study** | |
| --- | --- |
|  |  |
| Please specify if the patient completed the entire course of the study as specified in the protocol or discontinued the study:  Ο Completed  If continued, did patient withdraw consent after completing study?  Ο no Ο yes  If yes, date consent withdrawn: \|__\|__\| \|__\|__\| \|__\|__\|__\|__\|  d d m m y y y y  Ο Discontinued | |
| If discontinued, please specify **the most appropriate** reason for early termination: | |
|  | |
| Ο Adverse event^2^, please specify: ………………………………………………………………… | |
|  | |
| Ο Patient lost to follow up  Date of last contact with patient in this study: \|__\|__\| \|__\|__\| \|__\|__\|__\|__\|  d d m m y y y y | |
| Ο Patient withdrew consent  Date consent withdrawn: \|__\|__\| \|__\|__\| \|__\|__\|__\|__\|  d d m m y y y y  Reason: …………………………………………………………………….. | |
| Ο Investigator’s and/or physician’s decision  Date of decision: \|__\|__\| \|__\|__\| \|__\|__\|__\|__\|  d d m m y y y y  Reason: …………………………………………………………………….. | |
|  | |
| Ο Other reason, please specify:  ……………………………………………………………………………..……………………..  ……………………………………………………………………………..…………………….. | |

--------------------------------------------------------------------------------------

**END OF Case Report Form**

**THANK YOU FOR FILLING OUT THIS FORM**
